# Experience and Health-Related Behavior in Times of the Corona Crisis in Germany: An Exploratory Psychological Survey Considering the Identification of Compliance-Enhancing Strategies

**DOI:** 10.3390/ijerph18030933

**Published:** 2021-01-21

**Authors:** Virginia Deborah Elaine Welter, Naemi Georgina Eliane Welter, Jörg Großschedl

**Affiliations:** Institute for Biology Education, Faculty of Mathematics and Natural Sciences, University of Cologne, D-50931 Cologne, Germany; nwelter@uni-koeln.de (N.G.E.W.); j.grossschedl@uni-koeln.de (J.G.)

**Keywords:** COVID-19, health, prevention behavior, risk behavior, compliance

## Abstract

Despite the need for enduring preventive behavior in times of the COVID-19 pandemic, several counteracting behavioral reactions can be observed worldwide. Considering the grave consequences resulting from such health-related risk behavior, we carried out an online questionnaire study to identify personal characteristics that frame a motivational sketch of those who refuse to follow the pandemic-related preventive measures. Our results from a sample of *N* = 570 German participants already indicate obvious changes in relevant variables in the four-week investigation period during the pandemic’s initial phase (25 March to 22 April 2020). Above all, the willingness to take preventive measures decreased, as did the assessment of the pandemic’s severity. The perceived own vulnerability also turned out to be clearly age-dependent and, overall, our sample showed a negative affectivity deviating distinctively from a reference sample. These and other findings allow for identification of sources for public health interventions that can help to increase compliance with required prevention behavior, and thus, counteract COVID-19.

## 1. Introduction

The extent to which COVID-19 was able to spread all over the world brings upon extensive social and economic changes and restrictions [1,2,3,4,5]. The primary goal of counteractive public health strategies such as lockdown, containment, and social distancing is to prevent a dynamic, exponential spreading of the virus in the population, which would result in an overstretching of the healthcare system [3,5,6].

These restrictions of social contact instituted by law and monitored by police, and the restrictions under which permitted social contacts are placed, are at the disposal of personal discipline and compliance [6]. Thus, on an individual level, the challenge lies in comprehending and coping with general and personal health risks as well as to gain insight into the importance of a consistent implementation of the recommended or statutory instructions for hygiene practice and social interaction [6,7]. From an epidemiological perspective, hygiene practice and prohibitions on contact appear to be necessary to contain the COVID-19 pandemic, which is particularly dangerous for vulnerable people with chronic health problems, immunocompromised patients, and people over 65 years of age [3,5].

Shortly after the corresponding regulations came into force, many people did not seem to come to terms with preparing for such behavior changes and adjustments easily. Compliance was refused or corresponding information and the associated request was ignored, as documented by numerous press reports [8,9]. In the context of a pandemic, such ignorance or careless behavior is, however, capable of interfering with the life and health of other people and, above all, people who are at high risk, because third-party and self-protection are directly interdependent.

In view of such behavioral phenomena at the beginning of the COVID-19 pandemic in March 2020, we examined ad hoc what individual intrapsychic conditions and circumstances could be associated with this behavior and what sources for public interventions may exist in order to increase the likelihood of situation-appropriate behavior. Such interventions appear to be absolutely essential, especially taking into account the increasing number of antilockdown protests and COVID-19 deniers who aggressively stand out against necessary preventive restrictions [10,11,12,13,14,15,16]. At present, this noncompliance also affects the willingness to protect oneself and others from infection by making use of the vaccination against COVID-19. Counteracting these phenomena requires health promotion approaches that offer remarkable potential to empower people to search for, adequately process, and communicate health-related information as well as to make health-promoting behavioral decisions within their everyday life. At best, such approaches consider the recipients’ individual basic conditions (such as prior knowledge, possible misconceptions, media consumption habits, cultural background, motivational and personality characteristics) and use them in implementing target group-specific communication and information strategies. In times of the COVID-19 pandemic, these strategies can be used to encourage as many individuals as possible to engage in preventive behavior at home/in their families, at school/university/work, and in their communities and associations by acting as a role model and talking persuasively to others about how to prevent a further spread of COVID-19.

## 2. Theoretical Background

### 2.1. Health Beliefs and Behavior

Individuals can present a wide variety of behaviors in different areas of life—the same applies to their health. Such health behavior generally refers to all attitudes, habits, and deliberate actions of a person that have a positive or harmful effect on their health. Health-promoting behavior (e.g., healthy diet or regular exercise) usually protects and strengthens a person’s health, while behavior which is harmful to health (e.g., tobacco or alcohol consumption) is at least potentially associated with the risk of a weakening or endangering health [17,18]. Under conditions of the COVID-19 pandemic, individual health behavior manifests itself specifically in the way that the stressful and threatening situation associated with potential infection of oneself and others is reacted to. Specifically, this means, for example, whether and how information is obtained and processed in a targeted manner, and what hygiene practices (e.g., wearing face masks, frequent hand washing) and restrictions relating to personal interaction and public life (e.g., following the stay-at-home appeal) are included and implemented by every single person.

In the 1950s, the social-psychological Health Belief Model (HBM) was developed in the US in order to understand why people do not follow health prevention strategies or make use of medical checkups [19,20,21,22,23]. Later, the model was also applied to compliance with or adherence to prescribed medical regimens [24,25,26]. This approach is therefore directly analogous to our study’s motivational starting point. According to the HBM, preventive health behavior is favored by the following conditions [11,27,28,29,30]:

- Perceived severity and vulnerability: First, people perceive a general health risk, then take preventive measures to reduce this risk; for example, many people do sports to reduce the risk of overweight or cardiovascular diseases. In addition, the likelihood of preventive behavior is further increased if people consider themselves to be at specific risk, for example, because there is corresponding previous illness in their family.
- Perceived benefits of behavior modification and low barriers: Furthermore, if a certain preventive behavior is regarded as actually effective in minimizing personal risk, this again increases the probability of corresponding behavioral performance. Finally, potential barriers and the effort involved in preventive behavior should be assessed as low as possible in order to increase the likelihood of its realization.

Considering these theoretical assumptions, the HBM can be classified as an expectation-value model, assuming behavioral motivation to be the product of the value of an action’s expected consequences (emotional or rational) and the expectation to achieve these consequences by showing a certain behavior. In addition, the HBM meets the category of so-called fear appeal theories, which assume that people must first emphatically be confronted with their risk and shaken up in order to change their behavior. These two characteristics of the HBM offer decisive advantages in the context of research on health behavior in times of the COVID-19 pandemic. For example, a meta-analysis of 105 studies on the influence of fear appeals [31] showed that people who can be persuaded that they are seriously at risk of damages to their health show more preventive behavior. These results are directly related to the fact that such fear appeals have a particularly favorable effect on health behavior regarding novel situations in which little information is available. The current COVID-19 pandemic is such a situation. A further advantage of HBM relates to the fact that it focuses on factors that can be influenced comparatively easily and quickly (in contrast to, for example, some character traits that are considered to be stable and difficult to influence). Thus, public health interventions that can be derived from the HBM in the pandemic context could be thoroughly effective [26]. Therefore, we have chosen the HBM as the theoretical starting point of our study. However, since the weaknesses of the model—e.g., the nonconsideration of other relevant influencing factors—should not be ignored, we have also considered further constructs of other relevant theories and models that complement the components of the HBM and that have proven to be predictive for preventive health behavior as well (see Section 2.2).

### 2.2. Preventive Behavior in Times of the COVID-19 Pandemic

Transferred to the probability of preventive health behavior in dealing with the COVID-19 pandemic, the following conclusions regarding the HBM can be drawn:

1. In the case of COVID-19, its extensive health-damaging potential was recognized and communicated in its entirety. Based on the previous and still incomplete data situation, the mortality risk for COVID-19 is estimated to be several times higher compared to seasonal influenza [32].
2. Due to the extraordinarily high virulence of COVID-19, which is reflected in dynamic, exponential infection rates, the individual risk of becoming infected and facing an unpredictable course of disease is comparatively high [3,5,32].
3. The effectiveness of the preventive measures may well be confirmed, since flattened curves of the infection numbers could be observed after these measures came into force. The risk of a droplet or smear infection with COVID-19 seems to be drastically reduced, especially due to the social contact restrictions and strict compliance with hygiene practice [3,5,32].
4. Regarding the estimation of required individual effort in order to perform preventive behavior, two classes of preventive measures must be distinguished first: (a) those who ask to practice additional behavior and (b) those who ask to refrain from habitual behavior patterns. The first category includes relatively inexpensive and time-efficient measures (such as regular thorough hand washing, use of disinfectants, wearing face masks) and is therefore associated with comparatively little behavioral effort. Regarding the second category, from a physical point of view it can be stated that it is energetically favorable to refrain from doing something rather than to do it. Regardless of these advantages however, the ‘costs’ of such an omission must always be considered, which—in the case of social isolation for example—could again act as a health risk factor.

**Figure 1:**
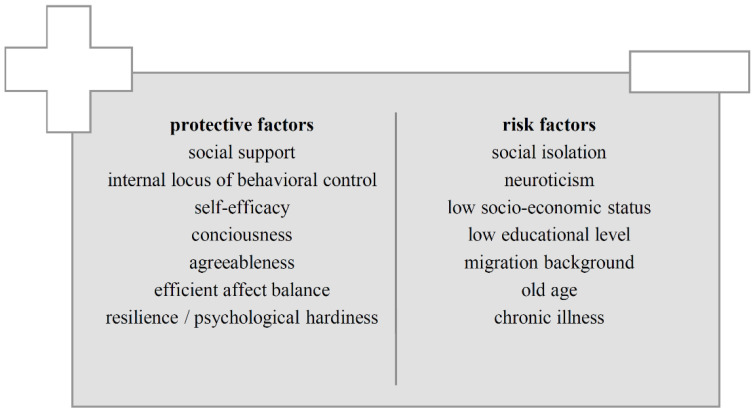
Important protective and risk factors relevant to health behavior beyond the Health Belief Model (HBM).

With this in mind, the question arises as to where behavior-relevant obstacles might be, which—as in the exemplifying newspaper reports [8,9]—cause so many people to gather in large groups ignoring the preventive guidelines. In particular, the individual willingness and ability to change or refrain from habitual behavior and patterns and to limit contacts that satisfy social and economic needs or even to do without them altogether (see aspect 4 of the above list) could be different depending on the assessment of one’s own vulnerability as well as the ratio of expected benefits to required effort. Such variations regarding an individual’s assessment can be caused by several factors. For example, the reproducible effect that people tend to incorrectly underestimate their own health risks compared to others [33] needs to be considered. Furthermore, such assessments also depend on the individually available information base, which, in turn, is influenced by multiple factors including prior knowledge, educational level, media consumption habits, and a lot more. Over the past decades, it has been shown that a substantial number of other influences on health behavior, such as demographic factors (e.g., gender, age, socioeconomic status) or psychological characteristics (e.g., personality traits, social interactions, behavioral locus of control, unconscious behaviors such as confirmatory information search), operate via changes regarding the components of the HBM or even act as supplementing but independent factors [34], tending to be more conducive or disadvantageous to preventive health behavior [35,36,37,38,39,40]. A selection of identified factors that seem to be decisive for individual health behavior and are therefore included in many relevant theories and models is shown in Figure 1 below.

Despite favoring the HBM as our study’s theoretical starting, we did not want to leave these other crucial factors unconsidered, so we operationalized them as additional variables (see Section 4.1). This approach seems to be evident, especially since several other studies on (non)compliance with COVID-19 containment measures have already shown that influencing factors not explicitly addressed by the HBM are decisive as well. For example, the Italian research group of Roma et al. [15] worked out the specific importance of self-efficacy, which mediates the relationship between perceived effectiveness of preventive measures and compliance with them. They found that an older age, a lower educational level, and low personality dysfunction are more likely to be associated with compliant behavior. Nivette et al. [13] as well as Chen and Chen [41] found identical results in their studies specifically focusing on Switzerland and China. In a further study regarding the impact of personality traits, Nofal et al. [14] were able to show that higher levels of consciousness, introversion, agreeableness, and openness to new experiences are significantly positively associated with compliance with containment measures. Furthermore, Pan et al. [42,43] carried out two studies showing that a realistic assessment of COVID-19′s severity as well as beliefs about the effectiveness of preventive measures depend decisively on the individually available information and knowledge base and the degree of trust in the political system. Conversely, they were able to show that misinformation, especially in cases of lack of knowledge and evaluation competency, is associated with a greater extent of concern and fear, and overall poor mental health. Since a negative affectivity balance could undermine health-promoting behavior in general [40], target group-specific public information campaigns on the COVID-19 pandemic seem to play an essential role in promoting compliance. Additionally, with specific regard to sociopsychological variables, Smith et al. [16] could show that increasing noncompliance with preventive behavioral measures is associated with reduced perception of social norms and social pressure. Such beliefs about what other people are doing and expecting others to do usually create a motivation to act similarly. In times of COVID-19, however, not only is the decrease in perceived social norms problematic, but it is quite conceivable that within the situation’s dynamics, social norms appear that have the reverse effect, as they are conducive to protest motivation.

Even if we could cite many more studies due to the intensive extent of research efforts, we limit ourselves at this point to the reported selection, since in our opinion it contains the essential findings. Almost all these research efforts, as well as our Germany-specific study, try to respond to the same central question about critical factors that can be used in the context of infection prevention. This question is of particular interest since appropriate authorities can detect and sanction the spread of a—perhaps not yet recognized—infection to others due to careless behavior at best by hindsight [44]. In order to prevent health damage in advance, the focus should be on preventive health promotion approaches until effective vaccines and treatments are available to the world’s population.

## 3. Objectives

Since the specific COVID-19 pandemic case is a topic which has, so far, not been extensively explored, our study was designed as an exploratory cross-sectional questionnaire study. In the first instance, our aim was to gain insight into how the COVID-19 pandemic affects selected central health-behavior-related aspects in the German population. Furthermore, we aimed at framing basic points of a sketch of the behavioral motivation of those noncompliant individuals who refuse to follow the measures taken to contain the COVID-19 pandemic. Both aims taken together should at least help to identify possible starting points for public health strategies that can help to increase compliance with required prevention behavior, and thus, counteract COVID-19.

Specifically, our study tried to answer the following research questions:

1. How does our sample assess the severity of COVID-19 and its own vulnerability to an infection in terms of the HBM? Is there an optimistic bias regarding the assessment of one’s own risk of infection compared to the risk of other people?
2. What and how many behavioral preventive measures are taken to protect oneself and others from an infection with COVID-19? Does the extent of the preventive behavior vary systematically between different subgroups of persons (e.g., between different ages or marital status) or are there relevant associations with other characteristics (e.g., affective or personality characteristics, satisfaction with information available, or the political management of COVID-19)?
3. Is there any evidence that either the extent of the preventive behavior shown and/or other variables relevant to health behavior changed during our four-week period of investigation?
4. What quality of affect balance can be determined in times of the COVID-19 pandemic compared to times before?
5. Based on exploratory correlational analyses between all captured variables relevant to health behavior, can specific central clusters be identified, which can serve as effective starting points for designing public health strategies to promote compliance in times of the COVID-19 pandemic?

## 4. Materials and Methods

### 4.1. Measure and Operationalization

Our online questionnaire covered a total of eight dimensions. In the following, the captured constructs/variables are explained in the order in which they were presented to the participants:

1. Sociodemographic characteristics: By means of eight self-constructed items, we collected data for the participants’ gender, age, nationality, marital status, postcode, household size, level of education, and professional status.
2. In order to quantify the characteristics of the components specified in the HBM, we captured the following COVID-19-related personal information by means of 10 self-constructed items:
  - Perceived severity of the COVID-19 pandemic (visual analogue scale, ranging from 0 = “not dangerous at all” to 10 = “extremely dangerous”);
  - Perceived own vulnerability to COVID-19 (visual analogue scale, ranging from 0 = “not at risk at all” to 10 = “extremely at risk”);
  - Possible affiliation to the COVID-19 high-risk group (dichotomous item answered by stating “yes” or “no”);
  - Presence of symptoms of a COVID-19 infection (cough, pyrexia, coryza, and/or sore throat; dichotomous items answered by stating “yes” or “no”) and, if applicable, virus screening including test results (adaptive question if COVID-19 symptoms were present);
  - Concerns about an own infection with COVID-19 as well as about infections of closely related persons (each designed as dichotomous item answered by stating “yes” or “no”);
  - Preventive measures taken (keeping distance, restricting contacts, following the stay-at-home-appeal, frequent hand washing, using disinfectants, and/or wearing face masks; dichotomous format answered by stating “taken” or “not taken”);
  - Satisfaction with the measures taken by politicians to contain the COVID-19 pandemic or reduce the number of new infections (categorical rating scale answered by stating “generally happy with it”, “more needs to be done”, “less needs to be done”, or “not sufficiently informed to rate this”), and, where appropriate, own suggestions for further measures (free text field);
  - Satisfaction with the media information regarding the COVID-19 pandemic (categorical rating scale answered by stating “generally happy with it”, “too little or too little reliable information”, or “confronted with too much information”);
  - Satisfaction with the with the measures taken by politicians to contain the economic consequences of the COVID-19 pandemic (categorical rating scale answered by stating “generally happy with it”, “more needs to be done”, “less needs to be done”, or “not sufficiently informed to rate this”).
3. Cognitive appraisal: The construct is based on the transactional stress theory by Lazarus and Folkman [45] and was captured using the Primary Appraisal Secondary Appraisal questionnaire (PASA) [46]. The 16 items of the PASA focus on the individually experienced strain within a stressful situation like the COVID-19 pandemic: The primary appraisal of such a stressor includes its perception as frightening (Subscale 1) and/or as challenging (Subscale 2), while the secondary appraisal takes the stressor-related self-concept of abilities (Subscale 3) and the locus of control (Subscale 4) into account. Overall, the questionnaire can therefore be used to relate the situation’s perception and evaluation to the assessment of individual available coping skills regarding this situation. The respective significance of this relationship in the context of the COVID-19 pandemic has already been shown by the exemplary reported findings by Roma et al. [15]. The four subscales are captured by four items each that should be assessed on a six-point rating scale. The mean is then calculated from the two mean values of the subscales of one dimension in order to obtain the values for the primary and secondary appraisal. The difference between the primary and secondary appraisal is finally used to calculate the total experienced strain as a *stress index*. The homogeneities of the primary scales in a reference sample were α = 0.61–0.83.
4. Personality: The *Big Five* represent the five cross-cultural and time-stable personality dimensions (1) *openness to new experience*, (2) *conscientiousness*, (3) *extraversion*, (4) *agreeableness*, and (5) *neuroticism* [47,48]. The importance of these personality traits in the context of the COVID-19 pandemic has already been shown by Nofal et al. [14]. To capture the *Big Five*, we used the short scale Big Five Inventory 10 (BFI-10) which comprises 10 items that should be assessed on a five-point rating scale [49,50]. The retest reliabilities of the five subscales in a reference sample were *r*_tt_ = 0.58–0.84. The decision for this short scale, which covers the *Big Five* dimensions with only two items each, was made in view of less strain on the participants, although the questionnaire is proven to be less reliable than other questionnaires comprising more items to measure the same personality constructs [51].
5. Social competence: As social support or perceived and communicated social norms are decisive influencing variables of individual health behavior [16,36,37,38,39,40], we have decided to capture selected aspects of social competence using 24 items from the Interpersonal Competence Questionnaire (ICQ) [52,53,54]. Overall, the questionnaire contains five scales with eight items each that should be assessed on a five-point rating scale. In our study, we decided to focus only on three subscales: (1) *negative assertion* (i.e., standing up for own rights and ability to criticize others), (2) *emotional support to others*, and (3) *effective handling of interpersonal conflicts*. The first omitted subscale “initiation of interactions and relationships” is largely redundant with the extraversion items of the BFI-10, and the second omitted subscale “disclosure of personal information” seemed to be irrelevant for the purpose of our study. The homogeneities of the three subscales in a reference sample were α = 0.77–0.84.
6. Reactance: According to Brehm [55] and Miron and Brehm [56], reactance describes an inner motivational resistance to perceived social influence, such as demands or prohibitions, which is experienced as restricting an individual’s freedom of behavior and control. In terms of behavior, reactance (*now more than ever* and/or *attraction of the forbidden*) is comparable to defiance as an active insistence on one’s own position. Fear appeals, which are increasingly used in times of the COVID-19 pandemic by decision-makers and the media, do not automatically result in a favorable effect regarding health behavior, but can even lead to reactance, especially if coping skills are not supported at the same time [28]. We used the unidimensional Reactance Scale [57,58], to capture reactance tendencies in our sample. The scale consists of 12 items that should be assessed on a four-point rating scale. The retest reliabilities of the five subscales in a reference sample were *r*_tt_ = 0.71.
7. Affect: The emotional situation of the test subjects in the last two weeks before participation in the study was captured using the Positive and Negative Affect Schedule (PANAS) [59,60]. The questionnaire comprises 20 items/mood-related adjectives that should be assessed on a five-point rating scale. When completing this questionnaire, subjects are asked to judge the adjectives in terms of how often they experienced a specific mood in the defined 14-day interval. Ten adjectives each represent the two dimensions of negative and positive affect, so it can be determined whether an individual’s affectivity was overall positive or negative and whether there are significant deviations compared with a reference sample from non-COVID-19 times. The homogeneity of the two subscales was α = 0.85 and 0.89 in a reference sample.
8. Resilience: The unidimensional Resilience Scale (RS-13) was used to capture the participants’ psychological resilience to stress and strain situations [61,62]. In contrast to the PASA items, which predominantly address situation-specific aspects, resilience rather describes a stable habitual tendency to react to stressors. As a short scale with 13 items that should be assessed on a seven-point rating scale, the RS-13 measures the extent to which stress experiences and negative emotions can be ‘cushioned’, to what extent people remain able to act, and whether they initiate necessary actions to cope with the stress/straining situation. The homogeneity of the scale was α = 0.90 in a reference sample.

Objectivity, factorial structure, and validity of the applied standardized scales described above (see bullets 3. to 8.) are supported by previous findings [46,49,50,52,53,54,57,58,59,60,61,62]. The reliability coefficients, which we determined for our sample, are shown in Table 1. Due to insufficient reliability, we had to exclude the consciousness and agreeableness subscales of the BFI-10 from further analyses.

### 4.2. Procedure

Using the Qualtrics Survey software (SAP America, Newtown Township, PA, USA) [63], we designed our online questionnaire as an open survey, which the participants could complete in about 20–30 min. In total, the questionnaire comprised 113 items, which were presented on 18 screens/pages using single- and multiple-choice or visual analogue scale formats. All questions were designed as forced-choice items, but all participants were free to select the answer option “I do not want to provide any information on this”. Additionally, we provided a back button, so all participants were able to change their answers. Usability and technical functionality had been tested before fielding the questionnaire.

The data collection took place over a four-week period at the beginning of the COVID-19 pandemic (25 March to 22 April 2020). The comparatively short-term recruiting of test subjects was accomplished by involvement of disseminators who were personally known to our research team. By e-mail and via social media, we invited all of our academic colleagues, students, relatives, and friends to take part in our survey and asked them in turn to advertise our study to everyone crossing their mind. So, altogether, we used a kind of chain-referral sampling. Additionally, our study was linked to the survey platform of the German Leibniz Institute for Psychology [64], where it was freely accessible for everybody visiting the open website. Participation was possible for all interested persons who were familiar with the German language, at least 18 years old, and equipped with the technical requirements (i.e., smartphone, PC or tablet, and internet connection) to complete an online survey.

Before filling in the questionnaire, all participants received a detailed written subject information in accordance with the current ethical guidelines laid down by the University of Cologne and the German Psychological Society [65], including the following information:

- aims and course of the investigation,
- absolute voluntariness of participation,
- possibility of dropping out of participation at any time,
- guaranteed protection of data privacy (collection of only anonymized data),
- possibility of requesting data cancelation at any time,
- no-risk character of study participation,
- contact information in case of any questions or problems.

According to the current version of the Declaration of Helsinki [66,67], written informed consent was obtained from all participants prior to the study.

After the participants declared their informed consent, they were asked to create an individual code to prevent multiple entries. This code consisted of the second character of their first name, the last character of their mother’s first name, the third character of their place of birth, and the first two numerals of their birthday. Within our data analysis, we did not identify any duplicate entries. After this procedure of creating the participant’s individual code, all subjects were informed of the importance of complete and honest answering of the questions. At the end of the questionnaire, this appeal was taken up a second time as a kind of manipulation check, asking “Have you answered all questions completely and as honestly and truthfully as possible?”. At this point, the participants were also able to state that they did not take part seriously and that their data should therefore not be further analyzed, this option was not taken up by any of the participants.

As an incentive to participate, a total of 10 Amazon vouchers, each worth €10 (about US $11.80), were raffled off among all test subjects who fully answered the questionnaire. Finally, all subjects had the opportunity to ask for a summary of our study’s results by sending an e-mail to our research team.

In total, 827 people participated in our online survey. Since we have decided to only analyze data sets that were 100% completely answered, 257 cases had to be excluded due to drop out of the survey before reaching the end of the questionnaire. Thus, the analyzed convenience sample amounted to a total of *N* = 570 cases, which corresponds to a completion rate of approximately 69%. View and participation rates cannot be reported for our survey, since we have decided to refrain from counting unique visitors (e.g., by determining their IP addresses) due to our institution’s data protection guidelines.

### 4.3. Sample

Of the *N* = 570 cases, more than two-thirds (70.5%) were female. Almost all (96.5%) stated that they had their primary residence in Germany, primarily in the Rhineland area in the west of Germany. The other participants with foreign domiciles mainly lived in areas bordering western Germany (e.g., the Netherlands), but were German nationals as well. The ages of subjects were between 16 and 88 years (*M* = 35.48, *SD* = 16.39).

**Figure 2:**
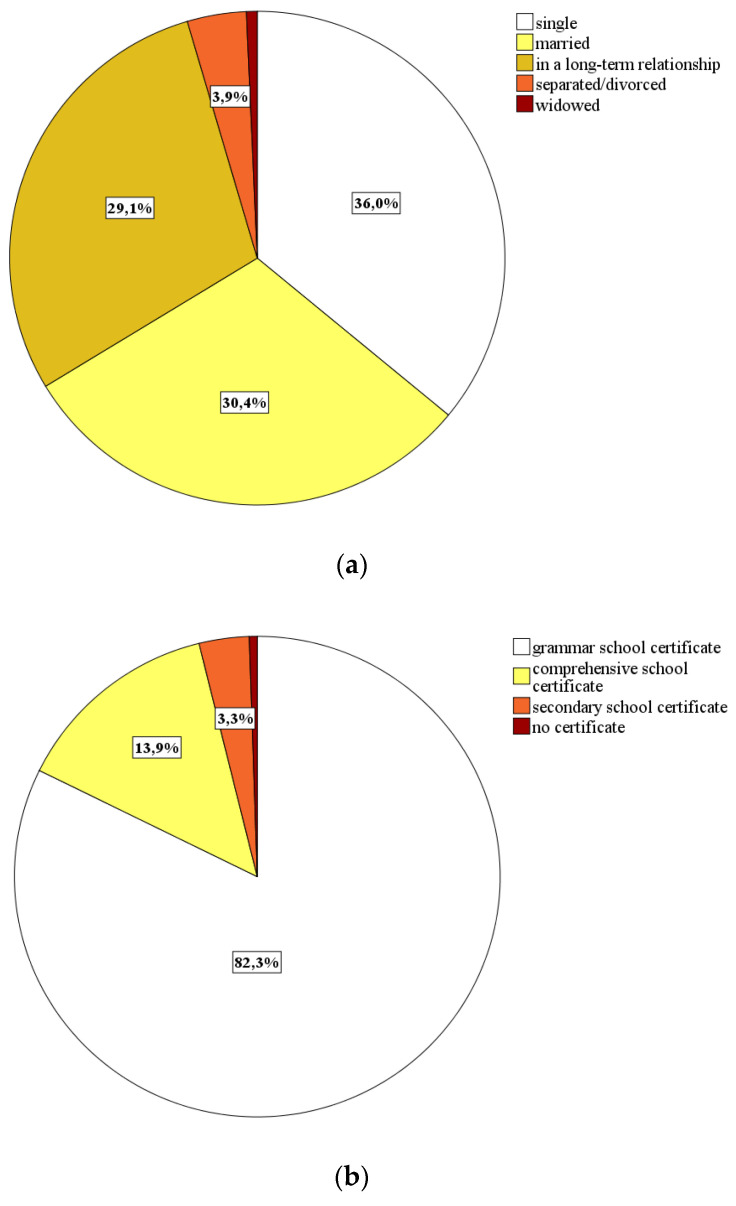

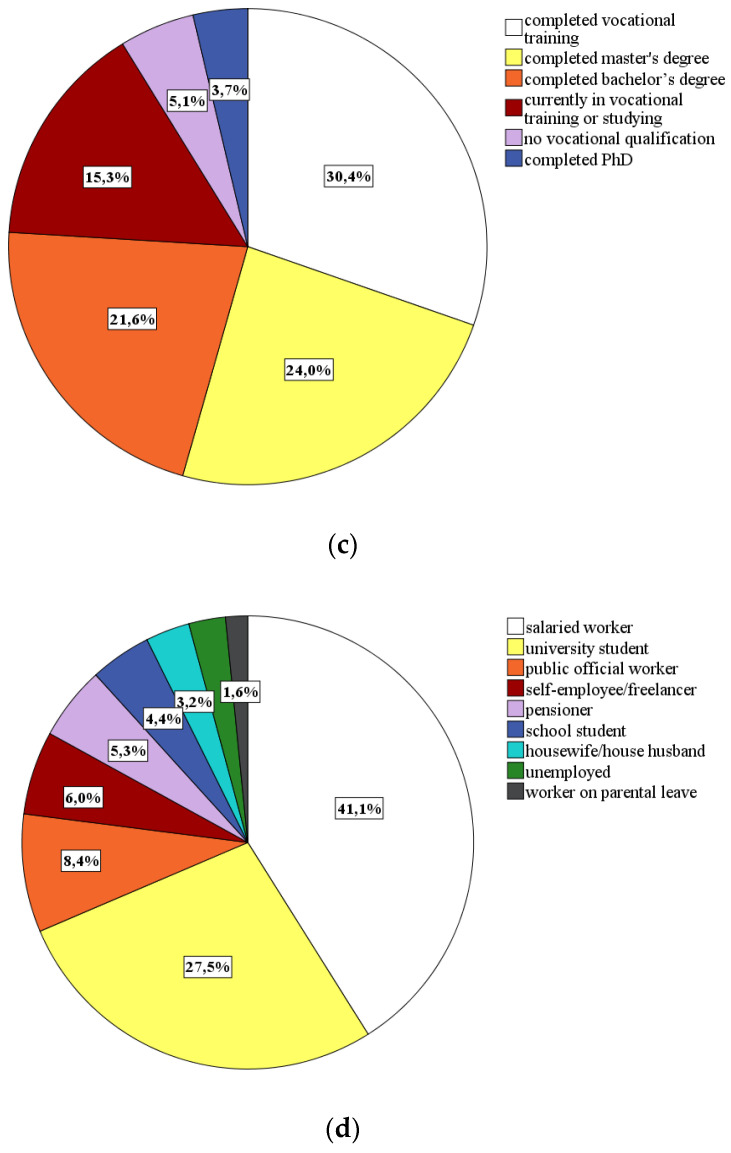
Sociodemographic sample characteristics (*N* = 570): (**a**) subjects’ marital status; (**b**) subjects’ school education levels; (**c**) subjects’ vocational training levels; (**d**) subjects’ current professional status.

Figure 2 provides information on further characteristics of our analyzed sample. While there is an approximately representative depiction regarding the German population’s marital status [68] (see Figure 2a), the samples’ distributions related to educational and vocational training levels as well as current professional status (see Figure 2b–d) are rather nonrepresentative [68]. This is most likely due to recruiting our participants predominantly in the university or academic context due to time pressure, while conducting our study during the pandemic’s initial phase. Since these nonrepresentative distributions are additionally associated with insufficient cell frequencies in some cases (e.g., *n* = 3 in the category *no school certificate*), we have refrained from carrying out group comparisons regarding these variables.

### 4.4. Statistical Methods

Statistical data analyses were carried out using the SPSS Statistics 25 software (IBM Corp., Armonk, NY, USA) [69]. The preparation of the data set included reliability analyses of the standardized scales used (see Table 1), scale and test score calculations according to the corresponding test manuals, analyses of sample characteristics (see Section 4.3), and calculation of descriptive results (see Section 5.1 and Section 5.2 ). After this, we checked for normal distribution of the captured variables as well as the calculated scale and test scores, which was at least satisfactory in nearly all cases. Given this, we carried out parametric Bravais–Pearson correlational analyses to test for potential relationships between the captured constructs (see Section 5.5). Potential group differences were analyzed using parametric and—in cases of violation of equality of variance and/or due to the level of measurement—nonparametric comparisons of means and analyses of variance (see Section 5.2 and Section 5.4). In addition, the four-week survey period was initially divided into two categories, *weeks 1 + 2* vs. *weeks 3 + 4*, for an analysis of possible differences due to the influence of the time course. For this, we created a new data set to compare the least common multiple (*n* = 82) of participants in both categories using random sampling (see Section 5.3). For all other analyses, the complete data set comprising *N* = 570 subjects was used.

## 5. Results

### 5.1. COVID-19-Related Personal Information and Assessments (Research Question 1)

Of the *N* = 570 subjects whose data was analyzed, *n* = 74 (13.0%) stated that they considered themselves to be at risk for severe courses of a COVID-19 infection due to their age being >65 years and/or chronic illness, *n* = 5 (0.9%) participants did not provide any information on this. Furthermore, *n* = 38 (6.7%) subjects were concerned about COVID-19 infecting themselves, and *n* = 238 (41.8%) were worried about close related persons that might be affected, so a typical optimistic bias seems to exist among our participants.

Additionally, *n* = 188 (33.0%) subjects reported suffering from at least one COVID-19-specific symptom (cough, pyrexia, coryza, and/or sore throat) [3,5] at the time of participation, *n* = 3 (0.5%) participants did not provide any information on this. Regarding related medical diagnosis, *n* = 30 (5.3%) participants stated that they had already been tested for a COVID-19 infection. Of these, *n* = 11 (36.7% out of *n* = 30) reported a positive test result, while *n* = 2 (6.7% out of *n* = 30) did not want to provide information on the test result.

Regarding the media information provided in the context of the COVID-19 pandemic, *n* = 316 (55.4%) participants stated that they were generally satisfied, *n* = 172 (30.2%) felt that they were confronted with too much information, and *n* = 82 (14.4%) judged the available information to be insufficient or not reliable enough.

Finally, *n* = 234 (41.4%) subjects indicated that they were generally satisfied with the measures taken by politicians to contain the economic consequences of the COVID-19 pandemic, *n* = 174 (30.5%) participants rated the measures to be inadequate, and *n* = 24 (4.2%) considered them to be too extensive. A total of *n* = 138 (24.2%) subjects did not feel sufficiently informed to be able to provide a satisfaction rating.

### 5.2. Preventive Behavior in Times of COVID-19 (Research Question 2)

**Figure 3:**
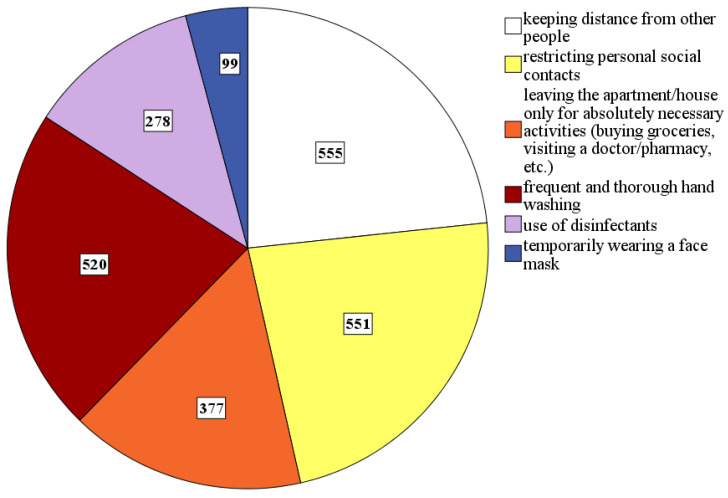
Number of *N* = 570 subjects who had taken specified preventive measures.

Concerning their preventive behavior, *n* = 569 (99.8%) subjects reported that they had taken at least one preventive behavioral measure to minimize the risk of a COVID-19 infection (see Figure 3).

Overall, *n* = 394 (69.1%) subjects stated that they were satisfied with the national recommended or statutory preventive measures to reduce the number of new infections, *n* = 42 (7.4%) participants felt that they were not sufficiently informed to be able to provide a satisfaction rating, and *n* = 29 (5.1%) participants considered the measures to be too extensive. The remaining *n* = 105 (18.4%) thought that additional measures should be taken:

- Rigorous curfew (i.e., permission to leave the house only for essential activities): *n* = 60 (57.1% out of *n* = 105);
- Closedown of all nonessential facilities, shops, and businesses: *n* = 59 (56.2% out of *n* = 105);
- Special opening times of the essential businesses for high-risk groups: *n* = 58 (55.2% out of *n* = 105);
- Provision of protective clothing and disinfectants for private use: *n* = 39 (37.1% out of *n* = 105);
- Border closure for all non-supply-related entry, exit, and transit traffic: *n* = 53 (50.5% out of *n* = 105);
- Rigorous ban on travel: *n* = 47 (44.8% out of *n* = 105);
- Others (especially obligatory use of face masks, volume testing, stronger checks on compliance with preventive measures): *n* = 11 (10.5% out of *n* = 105).

**Figure 4:**
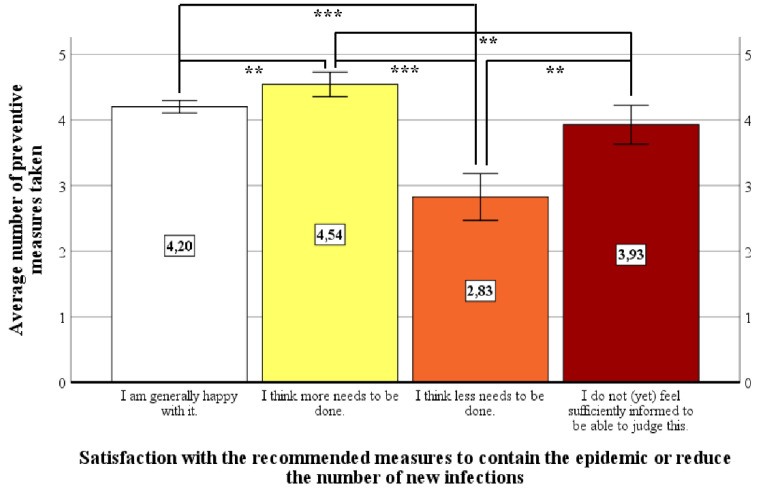
Differences in prevention behavior depending on satisfaction with the national recommended/statutory preventive measures (** = *p* < 0.01, *** = *p* < 0.001).

A nonparametric analysis of variance (Kruskal–Wallis-H test) was carried out to answer the question of whether or to what extent performed preventive behavior depends on the satisfaction with the national recommended or statutory preventive measures. In order to determine the average number of preventive measures taken, we first calculated a sum score for the maximum out of six possible preventive measures categories for each participant. These sum scores (*M* = 4.18, *SD* = 1.03) showed a sufficient range of variation and an appropriate Gaussian distribution so, finally, mean values could be calculated. Differences in the mean of preventive measures taken to reduce the risk of an infection with COVID-19 exist between all four groups based on their stated satisfaction, χ^2^(3) = 44.30, *p* < 0.001, d_Cohen_ = 0.56. The difference between the group that considered the measures to be too extensive and the other three groups is particularly clear. Even those subjects who stated that they did not feel sufficiently informed to be able to give a satisfaction judgement reported that they implemented more preventive behavior than those who rated the measures to be too extensive (see Figure 4).

**Figure 5:**
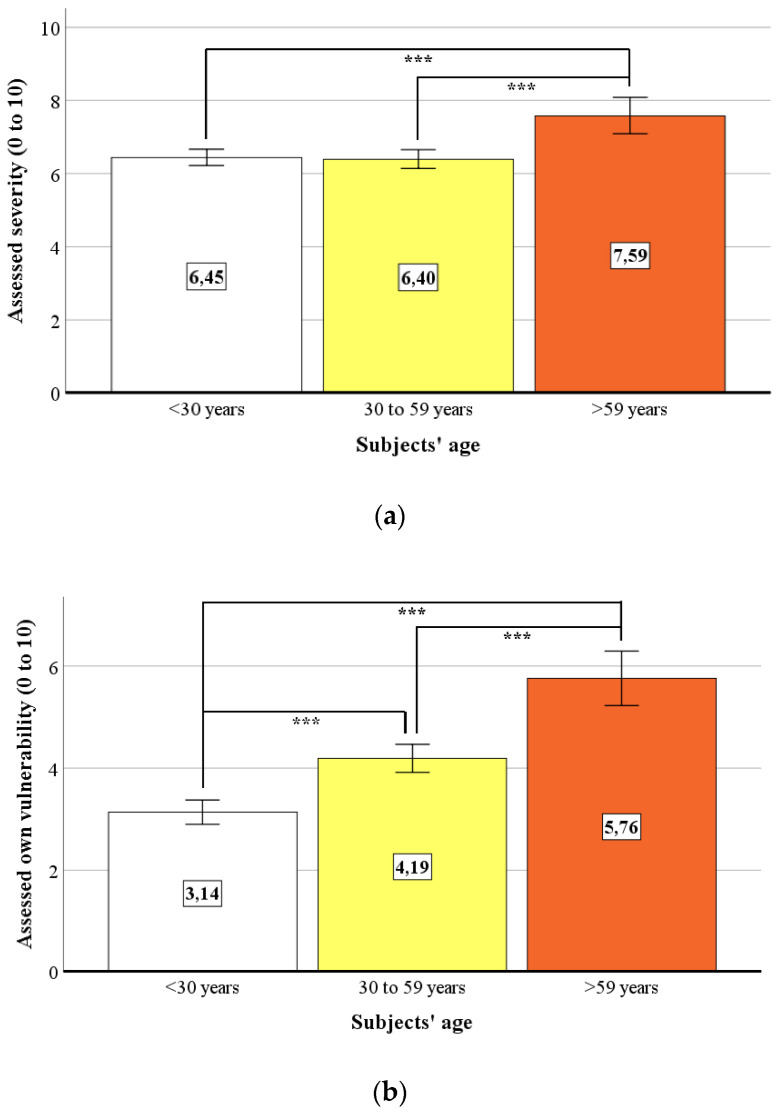
Age-dependent assessments of COVID-19: (**a**) Assessment of COVID-19′s severity (*** = *p* < 0.001); (**b**) assessment of subjects’ own vulnerability to COVID 19 (*** = *p* < 0.001).

Since several media reported that older people in particular have a comparatively high risk of developing a severe course of a COVID-19 infection [3,5], we wanted to investigate whether the HBM-based [19,20,21,22,23] assessments of the perceived severity of COVID-19, as well as the individual vulnerability and the mean number of preventive behavioral measures taken, differ significantly between different age groups. For this, we created a total of three age-related categories: <30 years (*n* = 292) vs. 30–59 years (*n* = 219) vs. >59 years (*n* = 58). Performed parametric and nonparametric (Kruskal–Wallis-H test) analyses of variance showed that the severity of COVID-19 as well as the subject’s own vulnerability were judged significantly differently depending on age *F*(2, 566) = 9.54, *p* < 0.001, d_Cohen_ = 0.35 (severity; see Figure 5a) and χ^2^(2) = 78.70, *p* < 0.001, d_Cohen_ = 0.79 (own vulnerability; see Figure 5b), but there were no significant differences in the mean number of preventive measures taken, *F*(2, 566) = 0.33, *p* = 0.72.

Furthermore, since Smith et al. [16] were able to show that prevention behavior varies depending on perceived social norms and social pressure, we wanted to examine whether there are similar trends in our German sample, depending on whether someone is living alone or with a partner. Therefore, it is quite plausible to assume that a continuing exposure to social influences within a partnership has a stronger influence on preventive behavior than if someone generally lives alone and his or her social contacts are additionally restricted by the pandemic situation. To answer this question, we have created two marital status-related categories: nonsingle (*n* = 339 subjects who stated being married or in a long-term relationship) vs. single (*n* = 231 subjects who stated being single, separated/divorced, or widowed). Performed nonparametric analyses of mean differences (Mann-Whitney-U test) showed that the subject’s own vulnerability to COVID-19 (U(*n*_1_ = 339, *n*_2_ = 231) = 30,082.50, *p* < 0.001, d_Cohen_ = 0.40) as well as the assessments of the pandemic situation as frightening (U(*n*_1_ = 339, *n*_2_ = 231) = 34,425.50, *p* < 0.05, d_Cohen_ = 0.21) or challenging (U(*n*_1_ = 339, *n*_2_ = 231) = 34,496.00, *p* < 0.05, d_Cohen_ = 0.20) were judged significantly differently depending on the subjects’ marital status. Additionally, there were small but significant differences regarding the mean number of preventive measures taken, U(*n*_1_ = 339, *n*_2_ = 231) = 3562.00, *p* < 0.05, d_Cohen_ = 0.17. All these differences found imply ‘disadvantages’ of the single group, i.e., not only do they rate their own vulnerability and the pandemic situation’s frightening and challenging character lower, but also take fewer preventive measures to protect themselves and others. Thus, it becomes apparent that the intensity of perceived social norms seems to be a crucial factor in our sample as well.

### 5.3. Influence of Time Course (Research Question 3)

An analysis of potential differences in the participants’ experience and behavior during the course of time was inspired by various media reports on corresponding accumulating stressors from the COVID-19 pandemic and is of particular interest to us. In order to quantify the influence of time course, the four-week survey period was initially divided into two categories, weeks 1 + 2 (25 March to 8 April 2020) vs. weeks 3 + 4 (9 April to 22 April 2020). A new data set was then created in which the total of *n* = 82 participants in the weeks 3 + 4 category were compared to a random sample of *n* = 82 from the total of *n* = 488 participants in the weeks 1 + 2 category. In order to control for potential baseline differences regarding background characteristics of these two samples, we performed Pearson’s χ^2^-tests which revealed no statistically significant deviations (*p*-values ranged from 0.08–0.87). Thus, the two samples were adequately comparable regarding their gender, age, marital status, school education level, vocational training level, current professional status, affiliation to the COVID-19 high-risk group, presence of COVID-19 symptoms, and satisfaction ratings relating to preventive measures, media information, as well as management of the pandemic’s economic consequences.

Regarding the preventive measures taken, performed cross table analyses showed that the use of disinfectants and face masks tended to increase. Conversely, compliance with other effective preventive measures such as keeping distance from other people or frequent and thorough hand washing decreased, although we cannot state statistically significant differences for these variables within the comparatively short survey period. With a *p*-value of precise 0.05, the decrease regarding following the stay-at-home appeal just hits the threshold of statistical significance. A de facto statistically significant decrease (*p* < 0.05) over the four-week study period resulted only in the restriction of personal social contacts (see Table 2).

A statistically significant decrease was also found for the assessment of the COVID-19 pandemic as frightening and/or challenging as well as for neuroticism. Corresponding to these findings, we found the perceived severity of the pandemic and the subject’s self-assessed own vulnerability to COVID-19 decreasing trendwise (see Table 3).

### 5.4. Affect Balance (Research Question 4)

In order to quantify the influence of the current COVID-19 pandemic on the population’s emotional situation, we compared the mean values for the dimensions of positive and negative affectivity to a reference sample from a validation study of the German version of the PANAS [70], which was examined before the COVID-19 pandemic and in times of no appreciable crises at all. The aggregated and pooled parameters reported for this reference sample enable a reliable estimation of the relative extent of possible differences in mean values relative to our sample by calculating Cohen’s d as effect size [71]. Compared to the reference sample (*N* = 349), significantly more negative affectivity was found among the participants of our COVID-19 pandemic sample. Regarding positive affectivity however, no significant differences to the reference sample could be determined (see Table 4).

### 5.5. Correlational Findings (Research Question 5)

Regarding the preventative behavior of the participants, correlational analyses showed significant relationships with the situational perception and cognitive evaluation of the COVID-19 pandemic as well as with personality traits, social competence, and the current affectivity. Overall, more preventive behavioral measures are taken as follows:

- The higher the severity of COVID-19 is assessed, *r*(568) = 0.21, *p* < 0.001;
- The higher one’s own vulnerability to COVID-19 is assessed, *r*(568) = 0.21, *p* < 0.001;
- The more the COVID-19 pandemic is perceived as frightening, *r*(568) = 0.23, *p* < 0.001;
- The more the COVID-19 pandemic is perceived as challenging, *r*(568) = 0.30, *p* < 0.001;
- The higher the current stress index is, *r*(568) = 0.21, *p* < 0.001;
- The more neurotic a person is, *r*(568) = 0.13, *p* < 0.01;
- The more open to new experiences a person is, *r*(568) = 0.14, *p* < 0.01;
- The better a person can emotionally support others, *r*(568) = 0.10, *p* < 0.05;
- The better a person can handle interpersonal conflicts, *r*(568) = 0.08, *p* < 0.05;
- The more negative affect a person is currently experiencing, *r*(568) = 0.15, *p* < 0.001.

**Figure 6:**
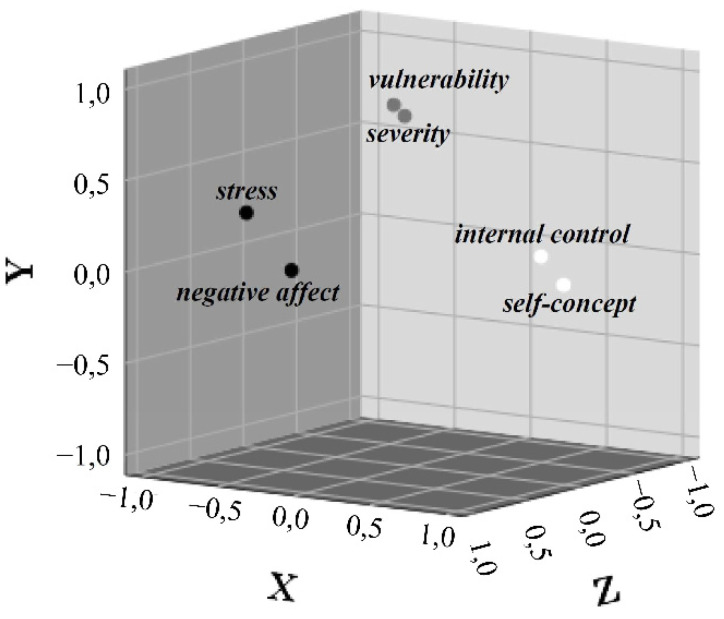
Component diagram in rotated space for the six central variables determined by factor analysis.

Furthermore, we have performed an exploratory correlation analysis between all captured constructs and their facets, whose results are presented in Table S1 (see Supplementary Materials). Due to the large number however, a detailed explanation of the individual correlational findings is not reasonable, so we will refer to condensed, comprehensive clusters at a glance. A grouping of the variables considered in our study via factor analysis allowed identification of those six central variables that showed the most numerous and strongest intercorrelations. The remaining variables were subsequently excluded from the analysis because they proved to be less relevant according to these criteria. We then simulated a Varimax-rotated three-dimensional arrangement of the determined six central variables and named the identified three clusters (1) hazard potential recognition, (2) personal coping skills, and (3) emotional response according to theoretical considerations and content overlaps (see Figure 6).

The selected representation condenses complex interrelationships: According to our analyses, a (in fact, socially desirable) COVID-19-related recognition of potential hazards seems to be associated with a reduction in self-assessed personal coping skills (correlations of the cluster-bound variables from *r*(568) = −0.11 to −0.16, *p* < 0.05 to <0.001). Furthermore, self-assessed personal coping skills again appear to be negatively associated with emotional reaction (correlations of the cluster-bound variables from r(568) = −0.61 to −0.72, *p* < 0.001), so a reduction in the self-concept of COVID-19-related abilities and the locus of control, for example, due to detection of potential hazards, is associated with an increase in experienced stress and negative affect. Finally, a greater stress experience and increased negative affectivity are associated with increased detection of the potential hazards of COVID-19 (correlations of the cluster-bound variables from r(568) = 0.16 to 0.25, *p* < 0.001).

## 6. Discussion

The main aims of our study were, on the one hand, to gain an exploratory overview of individual characteristics associated with COVID-19-related prevention behavior and, on the other hand, to identify starting points for health-promoting strategies to enhance individual compliance with it.

The data collected from *N* = 570 test subjects indicates that both the detection of COVID-19-related hazards and a negatively connoted emotional reaction to the pandemic situation are each positively correlated with compliance with recommended preventive measures. However, we also found that the self-reported number of implemented preventive measures already decreased from the third week of our study. The analysis of the time course’s influence showed that, in parallel, assessment of the pandemic’s severity and the subject’s perceived own vulnerability to COVID-19 as well as the assessment of the pandemic situation as challenging were reduced. Additionally, considering the decreasing frequency of recommended preventive behavior, this indicates a decrease in attention and/or taking the current situation seriously. Such a finding is in line with the motivational starting point of our study, regarding those people who actively refuse to implement epidemiological recommendations concerning their everyday behavior.

A more in-depth analysis of the correlative relationships between the captured variables provided evidence as to how these findings combine to frame a sketch of the behavioral motivation of these noncompliant refusers. According to the HBM [19,20,21,22,23] as well as our empirical data, it is plausible that a more extensive COVID-19-related detection of potential hazards goes hand in hand with increasingly practiced preventive behavior. Accordingly, it could be assumed that the refusers’ behavioral motivation could be attributed to a lack of information, which makes it difficult to adequately identify potential risks, since we were able to show that almost half of the participants felt insufficiently informed. On the other hand, or possibly also in addition, the preventive measures could be experienced as overwhelming, both due to the (largely socially isolating) conditions in which they live, as well as through no direct recognizable positive effect of the measures and thus the effectiveness of one’s own behavior.

The psychological dynamics of the currently increasing negative affect, which deviates distinctively from a reference sample, is also correlated with reactance, *r*(568) = 0.46, *p* < 0.001. This again reinforces the empirical observations that disciplined and considerate behavior is replaced more and more by active rejection and simultaneously by incorporating familiar behavior and patterns. If such a change in behavior does not affect the detection of potential hazards related to the pandemic, it can be expected that such comparatively risky behaviors will further intensify experienced stress and negative affect. Specifically, this means that if recognizing the pandemic’s severity on the one hand is generally associated with greater willingness to act in a more preventive way, but is also associated with increased negative connoted emotional reactions on the other hand, which in turn are associated with reactance, less reasonable behavior can be expected with increasing awareness of loss of internal control and self-efficacy. Conversely, for our result of a positive correlation between experienced stress and negative affect, on the one hand, and increasingly preventative behavior on the other hand, this would mean that it could also be a transient intermediate. It is conceivable that after an initial phase of compliance, overcoming the ‘first horror’ caused a decrease in the willingness to take preventive measures in the second half of our test period (from the beginning of April 2020). This may well have signalized the rise of virulent protests against preventive measures [8,9].

The development of such a reduction in the willingness to act reasonably from an epidemiological perspective can also be found in the current discussion on the so-called prevention paradox [72]. It claims that the effectiveness of a measure leads to the measure being considered unnecessary (even initially). The decisions made and lock-down measures taken by the government have had clear effects and, accordingly, the German healthcare system has not been overstretched so far.

In the same line of the consequent misjudgments “all clear” and “it was not that bad” lies the empirical lack of experience of both the pandemic and the effects of one’s own preventive behavior. Most people are initially confronted with COVID-19 as a media phenomenon of numbers and abstracts, dominated by experiencing massive restrictions and changes in their everyday life. In their immediate world of experience there are, in most cases, no willful confrontations with COVID-19 patients or the consequences of an infection. This situation does not fundamentally differ from comparable situations in other fields in which there is nothing directly experienced and/or the consequences of which one is not (yet) directly confronted with (e.g., particulate matter pollution, climate change). This lack of immediate personal experience of the actual problem and the effectiveness of one’s own behavior in this respect is understandably associated with disinterest, ignorance, and lack of motivation regarding required behavior [73].

The question of whether the so-called anti-lock-down and antimeasure protests reported worldwide these days are to be considered as a genuine part of the construct of interest to us cannot be decided on the basis of our data. The preventive measures are not only offensively, but also demonstratively disregarded in the context of these protests [8,9]. The behavioral motivation of some protesters will certainly be primarily based on regaining control over their economic and financial situation and/or their personal freedom. From an epidemiological perspective however, they simply expose themselves and others to an increased risk of getting infected during the mass events. The factual and, above all, media presence of this resistance is also inextricably linked to the question of the consequences of such presentations. It is, for example, possible that people who are already in two minds regarding the extent of preventive behavior question their pandemic-appropriate discipline/commitment and may even reject it. Such negative consequences could in turn be plausibly explained by the described multidirectional relationships between detection of potential hazards, personal coping skills, emotional reaction, and reactance.

In summary, the main problem seems to be that a detection of potential hazards would be conducive to increased practice of preventive behavior, which in turn, can counter the uncontrolled pandemic spread of COVID-19. However, this recognition of potential danger also undermines the perceived personal coping skills, what is exacerbated by the lack of experienced effectiveness concerning one’s own preventive behavior. Perceived low personal coping skills, in turn, are associated with negative connoted emotional reactions, which on the one hand go along with improved recognition of the hazard potential, but on the other hand are associated with reactance to an even greater extent. The crucial point is therefore which health-promoting strategy could be used to increase compliance in the population and, at the same time, to reduce experienced stress and negative affect.

### Health-Educational Sources for Enhancing Compliance with Preventive Behavior

Considering only the HBM [19,20,21,22,23], it would not be plausible to expect that there will be appreciable resistance or protests against epidemiological behavioral recommendations, so further variables need to be taken into account for an adequate explanation of the relevant phenomena and, if necessary, to positively influence them. Our study’s results and the previously discussed considerations allow for identification of potential sources for specific interventions that can help to increase compliance with epidemiologically required prevention behavior.

Our findings suggest that motivation and compliance regarding preventive behavior could be enhanced by strengthening the self-concept of (COVID-19-related) abilities, self-efficacy, and internal locus of control, because less experiences of stress and negative affect can be expected, which in turn are associated with reactance. However, since a negatively connoted emotional reaction is also conducive to a corresponding hazard potential detection, special attention must be paid to the simultaneous enhancement of a realistic perception of the pandemic’s severity when selecting appropriate intervention strategies. To this effect, a targeted advertisement for psychology-oriented information and, above all, a communication campaign could be helpful. Corresponding implementation of such a campaign should be as broad as possible in terms of form, content, and media. Specifically, such a concept should address as many people as possible from different target groups in a well-considered manner. This also means answering the question in which way and via which channels one can most likely reach a specific target group. In our study, we were able to show that—contrary to earlier findings [74,75]—openness to new experiences seems to be particularly salient for practicing preventive behavior. This personality trait is generally distinctive in young adults [76]. Therefore, it seems worth considering that it could be important to specifically address younger people (<30 years) as part of their school/university studies as well as via their preferred media formats (especially social media).

Another problem, namely, that apparently or factually contradictory information is circulating in this novel situation, can only be countered by a public institution continuously reporting and commenting on the situation via different media. Particularly suitable for this are—also age- and target group-specific—processed, structured, and factually presented information and justifications using understandable terms, numbers, and diagrams. In this context, particular attention should be paid to avoiding misunderstandings, such as the widespread misconception that only the elderly are at risk of severe courses of a COVID-19 infection. Misunderstandings regarding the behavior appropriate to a pandemic could also be avoided through a well-considered use of the role model function of certain people. Here, it is also important to identify target group-specific role models first before requesting them to participate in corresponding campaigns. It is an uncomplicated and effective form of social control with a steering function, in that it is easier to show others what to do, rather than just verbally communicating it.

In addition, fear and uncertainty could be reduced, for example, through de facto advertising campaigns conventionalizing characteristic objects (face masks, disposable gloves, etc.) and/or gestures (greeting by touching the feet, etc.) into gadgets and/or badges which occur in everyday life. In exactly the same manner, conventional brand advertising also seeks to enforce certain habits in order to make products become an integral part of everyday life and the habitual behavior of consumers. In addition, a corresponding repetition and variation of core messages (e.g., in the form of posters or billboards) in the sense of the so-called Mere Exposure Effect promises habitual familiarity and, consequently, a tendency towards a more positive attitude towards the advertised ‘product’.

Further efforts should address the lack of immediate experience of COVID-19 and the consequences of an infection. Here, it is possible to work with pictures and/or short films (via TV, social media, posters, etc.), comparable to the BE SMART! campaign for traffic safety in Germany [77]. Individual fates of COVID-19 patients, high-risk patients, doctors and caregivers at the limits of their strength, etc. could be emphatically presented in order to specifically impart a physiognomy to the abstractly perceived pandemic threat.

In connection with this however, the problem of the lack of noticeable effects of one’s own prevention behavior arises. In particular, the individual and interpersonal behavioral requirements can be clearly associated with expected positive effects. Although these effects cannot be experienced directly, they should be continuously reported and could be made a subject of discussion in order to keep people aware of what is important, particularly in so-called lock-up phases, in which prevention-related restrictions are somewhat relaxed due to comparatively low numbers of new infections. In this regard, for example, employers—especially public ones—as well as educational institutions (schools, universities, etc.) could be encouraged to take part in creating a permanent awareness of the general threat situation beyond the implementation of statutory measures and without increasing fears. This could possibly be realized via regular briefings, asking for suggestions from employees/students, and/or a daily report on infected persons and possibly deaths in the immediate vicinity of everyone.

The effects of positive reinforcement in terms of operant conditioning could be used in some cases too, for example, via giving encouragements that light up when nearing electric traffic signs. In this context, it would also be conceivable to provide reinforcing feedback on prevention-oriented movement profiles (mainly staying at home, avoiding large crowds, etc.), which could, for example, be recorded using an app in accordance with privacy regulations. Such an app could also be implemented in the bonus campaign of statutory health insurance, so the insurant could receive another pecuniary incentive to perform the desired behavior.

Finally, a stronger participation of the population in regulated public–political decisions should be considered, especially to strengthen compliance and stabilize self-concept skills, subject to the condition that equivalent options for action are available. Appropriate participation or the conviction that one can have a say in relevant areas of life increases one’s personal motivation to act by strengthening one’s self-efficacy and internal locus of control [78,79,80]. Studies on the shared-decision-model have shown that participation stimulates an increased search for health-related information [81] and can also lead to favorable health behavior [82,83,84,85]. In other respects, such a ‘regulated’ empowerment could also be suitable to undermine the motivation for protest/boycott participation, which, to a large extent, may be based on the motive of a compensation for feelings of helplessness and external self-determination [86,87].

## 7. Limitations and Conclusions

Our study is subject to three notable limitations:

1. The first one relates to the usual methodological aspects of a self-selected sample and the resulting lack of representativity, which is associated with decreased external validity. As a result of the recruitment of subjects primarily in an academic context, the proportions regarding the educational level and professional status were distorted compared to the total population. We were therefore unable to carry out group comparisons in this regard, which focus on the assessment of the pandemic and preventive measures taken, although we had intended to do so. In addition, due to the design of the study as an online survey, fewer older than younger subjects could be addressed, since only 67% of all people at the age of >65 years have internet access at all (in contrast to 96–99% of all people at the age of <65 years) [88]. Correspondingly, our results can be interpreted as a valid indication of certain processes in the population, but they should not be transferred offhand and to the total population.
2. Secondly, some subscales of our questionnaire showed low or even insufficient reliability coefficients. A total of five subscales (three of the PASA and two of the BFI-10) of the overall 16 standardized subscales used, showed reliability coefficients only between 0.64 and 0.69. Nevertheless, we think that this did not affect internal validity excessively, since the comparatively large sample as well as the partly convergent other scales, which show good to excellent reliability coefficients, and whose results are in line with those of the less-reliable scales, compensate most of this particular lack of reliability. However, considering the two BFI-10 subscales *consciousness* and *agreeableness*, the reliability was completely insufficient (<0.60), so we had to exclude these subscales from further statistical analyses to avoid impairment of our conclusions’ validity. Of course, such an exclusion is always associated with a loss of information. Although the BFI-10′s test authors reported good psychometric properties within the questionnaire’s validation for a German sample, other studies recently carried out also seem to consistently meet reliability problems, especially with regard to the two subscales *consciousness* and *agreeableness* [89,90]. This indicates some shortcomings within the specification of the original measurement model and/or the test authors’ validation procedure. Future studies specifically focusing on the influence of personality traits on individual health behavior should therefore use other instruments to assess the Big Five, which offer better psychometric properties.
3. Thirdly, both the sample bias and the reliability problems made it impossible for us to give extensive valid subgroup-specific recommendations regarding health promotion strategies to enhance compliance with preventive measures. Particularly, with regard to behavior-relevant competencies related to compliance with preventive measures during the COVID-19 pandemic, other relevant studies pointed out the differential effectiveness of implemented campaigns regarding communication of information and behavioral instructions. This means that in addition to culture-specific differences, attention must be paid to demographic, motivational, affective, cognitive, psychosocial, political, and religious characteristics of different target groups as well, as these can have a decisive influence on the reception and interpretation of information [42,43,91]. Accordingly, it seems plausible that certain behavioral appeals can cause the desired behavior by acting as effective social norms (especially in collectivistically oriented cultures like many Arab and Asian countries) or the same appeals rather tend to cause reactance (especially in individually oriented cultures like western industrial nations) [91,92]. In addition to such fundamental problems regarding the reception of information, there are many other aspects affecting the specific design of communication. For example, the complexity of the information provided should be aligned with the educational level and language barriers should be taken into account as well in order to simply ensure comprehensibility [42,43,91]. Due to these factors, it is conceivable that people who live in problem districts, or the rural population compared to metropolitans need different messages in order to address them effectively. The accessibility of different target groups in terms of their preferred sources of information must also be considered. For example, younger people are more likely to use social media, while older people are more likely to use traditional print or broadcast media [68]. Another decisive factor affecting the acceptance of information is who is providing the information. Different population subgroups differ in who they perceive as a peer or authority, so social norms intended to implement by communication can have differential effects [91,92]. This differential effectiveness of information management should be examined systematically in longitudinal studies in order to be able to implement effective campaigns without delay in the future.

Nevertheless, our study allowed for taking a snapshot of behavioral tendencies which, even in smaller frames and contexts, make further investigations worthwhile. Especially, with regard to the development of empirically based procedures for implementing measures and health-promoting interventions, our results enable a better understanding of the underlying mechanisms and can therefore enhance the effectiveness of such measures and interventions as well as the individual’s compliance. The personal behavior-related adjustments which may be necessary due to a global threat scenario could be paved by appropriate general preparation and preventive knowledge transfer, keeping in mind the compliance that is required in those situations. The need for such a health-promoting approach aiming to reduce resistance and barriers, and to strengthen rational deliberateness, seems to be made clear by the pandemic situation.

Finally, it should be noted that our investigation was carried out against the specific background of Germany. In this regard, it must be considered that Germany’s political pandemic-related decisions were largely based on scientific findings or medical expert advice [93,94]. In addition, Germany offered very good preconditions in terms of the capacity and quality of its healthcare system. However, the advantages of Germany regarding the COVID-19-related infection rates and deaths, which have been emphasized many times and also internationally, are exclusively revealed compared to countries that have so far had little success in managing the pandemic (e.g., Great Britain, Brazil, or the US). If Germany, on the other hand, is compared to countries like China, South Korea, Taiwan, or Vietnam, it becomes obvious that the Asian countries have consistently performed a lot better in counteracting COVID-19 [95]. Since the outbreak of the second wave at the end of September 2020, the infection rates in Germany have increased rapidly. At the end of October, the number of infected people per million in South Korea was only about 1.3% of the German counts, and in China, Taiwan, and Vietnam, it was about only 0.7% [96]. Even if a differential analysis of the differences between countries regarding the pandemic situations as well as the population’s behavior tendencies is not our focal point of interest, such a comparison of countries with varying degrees of success in the pandemic’s containment can be of global interest in terms of crucial conditions for success and effective measures that can be derived from them.

## Figures and Tables

**Table 1:** Detailed internal consistencies of the used standardized instruments for the present sample.

Instrument	Subscale	Reliability
PASA	assessment as frightening	0.80 ^1^
	assessment as challenging	0.69 ^1^
	self-concept of COVID-19-related abilities	0.66 ^1^
	COVID-19-related locus of control	0.64 ^1^
BFI-10	openness to new experience	0.64 ^2^
	conscientiousness	0.52 ^2^
	extraversion	0.72 ^2^
	agreeableness	0.26 ^2^
	neuroticism	0.64 ^2^
ICQ	negative assertion	0.84 ^1^
	emotional support to others	0.88 ^1^
	effective handling of interpersonal conflicts	0.79 ^1^
Reactance Scale	—	0.80 ^1^
PANAS	positive affectivity	0.83 ^1^
	negative affectivity	0.83 ^1^
RS-13	—	0.89 ^1^

^1^ Cronbach’s α; ^2^ Spearman–Brown coefficient. PASA—Primary Appraisal Secondary Appraisal questionnaire; BFI-10—Big Five Inventory 10; ICQ—Interpersonal Competence Questionnaire; PANAS—Positive and Negative Affect Schedule; RS-13—Resilience Scale.

**Table 2:** Differences between the first and second half of the four-week investigation period with regard to preventive measures taken.

Preventive Measure	*n*_taken_^1^	Comparison	*n*_obs_^2^	*n*_exp_^3^	χ^2^-Test	*p*	φ
Keeping distance	160	Weeks 1 + 2	81.0	80.0	χ^2^(1) = 1.03	0.31	
		Weeks 3 + 4	79.0	80.0			
Restricting social contacts	156	Weeks 1 + 2	81.0	78.0	χ^2^(1) = 4.73	<0.05	−0.17
		Weeks 3 + 4	75.0	78.0			
Following the stay-at-home appeal	104	Weeks 1 + 2	58.0	52.0	χ^2^(1) = 3.79	0.05	
		Weeks 3 + 4	46.0	52.0			
Washing hands	152	Weeks 1 + 2	78.0	76.0	χ^2^(1) = 1.44	0.23	
		Weeks 3 + 4	74.0	76.0			
Using disinfectants	81	Weeks 1 + 2	36.0	40.5	χ^2^(1) = 1.98	0.16	
		Weeks 3 + 4	45.0	40.5			
Using face masks	27	Weeks 1 + 2	10.0	13.5	χ^2^(1) = 2.17	0.14	
		Weeks 3 + 4	17.0	13.5			

^1^*n*_taken_ = number *n* of 164 participants who reported having taken the specified measure; ^2^ *n*_obs_ = observed number; ^3^ *n*_exp_ = expected number under conditions of equal distribution.

**Table 3:** Differences between the first and second half of the four-week investigation period regarding selected aspects of the perception and assessments of the COVID-19 pandemic.

Aspects of the Perception and Assessments of the COVID-19 Pandemic	Comparison	*n* ^1^	*M* ^2^	*SD* ^3^	*t*-Test	*p*	d_Cohen_
Perceived severity	Weeks 1 + 2	82	6.62	2.01	*t*(162) = 1.96	0.05	
	Weeks 3 + 4	82	6.04	1.80			
Perceived own vulnerability	Weeks 1 + 2	82	3.84	2.30	*t*(162) = 1.83	0.07	
	Weeks 3 + 4	82	3.23	1.96			
Challenge assessment (PASA) ^4^	Weeks 1 + 2	82	4.83	0.81	*t*(162) = 2.35	0.02	0.37
	Weeks 3 + 4	82	4.54	0.78			
Neuroticism (BFI-10) ^5^	Weeks 1 + 2	82	3.05	1.00	*t*(162) = 2.10	0.04	0.33
	Weeks 3 + 4	82	2.74	0.90			

^1^*n* = number of participants; ^2^ *M* = mean; ^3^ *SD* = standard deviation; ^4^ PASA—Primary Appraisal Secondary Appraisal questionnaire; ^5^ BFI-10—Big Five Inventory 10.

**Table 4:** Differences in the participants’ positive and negative affectivity compared to a representative reference sample.

Affectivity Dimension	Comparison	*n* ^1^	*M* ^2^	*SD* ^3^	d_Cohen_
Positive affectivity	Reference sample	349	27.35	6.38	0.15
	Analysis sample	570	28.35	6.57	
Negative affectivity	Reference sample	349	14.67	5.19	1.01
	Analysis sample	570	20.78	6.55	

^1^*n* = number of participants; ^2^ *M* = mean; ^3^ *SD* = standard deviation.

## Data Availability

The data presented in this study are available on request from the corresponding author. The data are not publicly available due to privacy.

## Supplementary Materials

The following are available online at https://www.mdpi.com/1660-4601/18/3/933/s1, Table S1: Results of the exploratory correlation test between the constructs and their facets.
