# Supplementary material for: Experience and Health-Related Behavior in Times of the Corona Crisis in Germany: An Exploratory Psychological Survey Considering the Identification of Compliance-Enhancing Strategies
Source: Int J Environ Res Public Health. 2021 Jan 21;18(3):933. doi: 10.3390/ijerph18030933 (PMC7908557; doi:10.3390/ijerph18030933)
Supplement: Supplementary file 1 [file ijerph-18-00933-s001.pdf]

Table S1. Results of the exploratory correlation test between the constructs and their facets.

	1a	1b	1c	1d	1e	2a	2b	2c	2d	2e
**1a COVID-19 as challenging (PASA)**	–									
**1b COVID-19 as frightening (PASA)**	.60 ***	–								
**1c Self-concept of abilities (PASA)**	-.38 ***	-.25 ***	–							
**1d Locus of control (PASA)**	-.13 **	-.13 **	.36 ***	–						
**1e Stress index (PASA)**	.73 ***	.72 ***	-.72 ***	-.61 ***	–					
**2a Extraversion (BFI-10)**	-.05	.02	.15 ***	.04	-.08	–				
**2b Agreeableness (BFI-10)**										
**2c Conscientiousness (BFI-10)**										
**2d Neuroticism (BFI-10)**	.21 ***	.18 ***	-.28 ***	-.21 ***	.32 ***	-.18 ***			–	
**2e Openness (BFI-10)**	-.02	.04	.15 ***	.09 *	-.08	.14 **			-.01	–
**3a Negative assertion (ICQ)**	-.07	-.04	.23 ***	.09 *	-.16 ***	.19 ***			-.27 ***	.09 *
**3b Emotional support (ICQ)**	.04	.13 **	.15 ***	.07	-.02	.22 ***			-.03	.17 ***
**3c Managing conflicts (ICQ)**	.00	.08	.22 ***	.10 *	-.09 *	.15 ***			-.26 ***	.18 ***
**4 Reactance (Reactance scale)**	-.05	-.12 **	-.04	.00	-.05	-.03			.01	.04
**5a Positive affectivity (PANAS)**	-.20 ***	-.14 **	.40 ***	.23 ***	-.35 ***	.21 ***			-.27 ***	.15 ***
**5b Negative affectivity (PANAS)**	.38 ***	.33 ***	-.39 ***	-.18 ***	.46 ***	-.07			.41 ***	-.01
**6 Resilience (RS-13)**	-.05	.05	.25 ***	.15 ***	-.14 **	.22 ***			-.37 ***	.22 ***
**7 Preventive behavior**	.23 ***	.30 ***	-.07	.01	.21 ***	-.03			.13 **	.14 **
**8a Severity of COVID-19**	.32 ***	.41 ***	-.11 *	-.13 **	.25 ***	-.09 *			.11 **	.03
**8b Vulnerability to COVID-19**	.24 ***	.30 ***	-.16 ***	-.01	.25 ***	-.13 **			.17 **	-.04

Continuation Table S1

	3a	3b	3c	4	5a	5b	6	7	8a	8b
**3a Negative assertion (ICQ)**	–									
**3b Emotional support (ICQ)**	.27 ***	–								
**3c Managing conflicts (ICQ)**	.30 ***	.50 ***	–							
**4 Reactance (Reactance scale)**	.05	-.10 *	-.21 ***	–						
**5a Positive affectivity (PANAS)**	.27 ***	.16 ***	.20 ***	.05	–					
**5b Negative affectivity (PANAS)**	-.18 ***	-.05	-.17 ***	.14 **	-.27 ***	–				
**6 Resilience (RS-13)**	.35 ***	.32 ***	.38 ***	-.05	.41 ***	-.31 ***	–			
**7 Preventive behavior**	.03	.10 *	.08 *	-.02	.03	.15 ***	-.01	–		
**8a Severity of COVID-19**	.00	.11 *	.01	-.04	-.05	.17 ***	.04	.21 ***	–	
**8b Vulnerability to COVID-19**	-.03	-.06	-.06	-.02	-.16 ***	.16 ***	-.10 *	.21 ***	.45 ***	–

Annotation. Parametric correlations according to Bravais-Pearson were calculated; grey highlighted cells = variables were excluded from analyses due to lack of reliability; *** = $p < .001$, ** = $p < .01$, * = $p < .05$; 1a = assessment of the pandmie as chellenging, 1b = assessment of the pandmie as frightening, 1c self-concept of COVID-19-related abilities, 1d = COVID-19-related locus of control, 1e = COVID-19-related stress experience, 3a = assertion of personal rights and ability to criticize others, 3b = emotional support to others, 3c = ability to handle interpersonal conflicts effectively, 7 = average number of preventive behavioral measures taken to reduce the risk of infection, 8a = perceived severity of COVID-19 in terms of the HMB, 8b = perceived own vulnerability to COVID-19 in terms of the HBM.
